# Bistable Functions and Signaling Motifs in Systems Chemistry: Taking the Next Step Toward Synthetic Cells

**DOI:** 10.1021/acs.accounts.4c00703

**Published:** 2025-01-22

**Authors:** Indrajit Maity, Nathaniel Wagner, Dharm Dev, Gonen Ashkenasy

**Affiliations:** † Department of Chemistry, Ben-Gurion University of the Negev, Be’er Sheva 84105, Israel

## Abstract

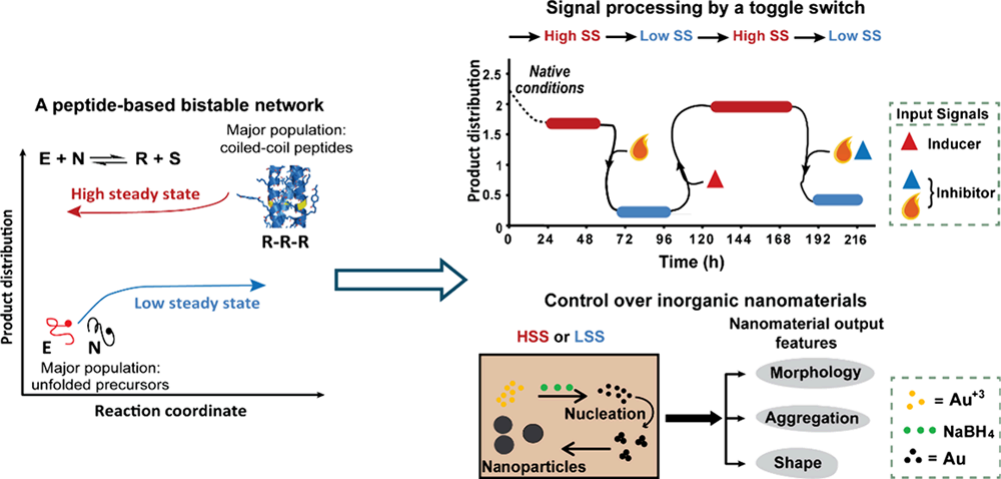

A key challenge in modern chemistry research is to mimic life-like functions using simple molecular networks and the integration of such networks into the first functional artificial cell. Central to this endeavor is the development of signaling elements that can regulate the cell function in time and space by producing entities of code with specific information to induce downstream activity. Such artificial signaling motifs can emerge in nonequilibrium systems, exhibiting complex dynamic behavior like bistability, multistability, oscillations, and chaos. However, the *de novo*, bottom-up design of such systems remains challenging, primarily because the kinetic characteristics and energy aspects yielding bifurcation have not yet been globally defined. We herein review our recent work that focuses on the design and functional analysis of peptide-based networks, propelled by replication reactions and exhibiting bistable behavior. Furthermore, we rationalize and discuss their exploitation and implementation as variable signaling motifs in homogeneous and heterogeneous environments.

The bistable reactions constitute reversible second-order autocatalysis as positive feedback to generate two distinct product distributions at steady state (SS), the low-SS and high-SS. Quantitative analyses reveal that a phase transition from simple reversible equilibration dynamics into bistability takes place when the system is continuously fueled, using a reducing agent, to keep it far from equilibrium. In addition, an extensive set of experimental, theoretical, and simulation studies highlight a defined parameter space where bistability operates.

Analogous to the arrangement of protein-based bistable motifs in intracellular signaling pathways, sequential concatenation of the synthetic bistable networks is used for signal processing in homogeneous media. The cascaded network output signals are switched and erased or transduced by manipulating the order of addition of the components, allowing it to reach “on demand” either the low-SS or high-SS. The pre-encoded bistable networks are also useful as a programming tool for the downstream regulation of nanoscale materials properties, bridging together the Systems Chemistry and Nanotechnology fields. In such heterogeneous cascade pathways, the outputs of the bistable network serve as input signals for consecutive nanoparticle formation reaction and growth processes, which–depending on the applied conditions–regulate various features of (Au) nanoparticle shape and assembly.

Our work enables the design and production of various signaling apparatus that feature higher complexity than previously observed in chemical networks. Future studies, briefly discussed at the end of the Account, will be directed at the design and analysis of more elaborate functionality, such as bistability under flow conditions, multistability, and oscillations. We propose that a profound understanding of the design principles facilitating the replication-based bistability and related functions bear implications for exploring the origin of protein functionality prior to the highly evolved replication–translation–transcription machinery. The integration of our peptide-based signaling motifs within future synthetic cells seems to be a straightforward development of the two alternating states as memory and switch elements for controlling cell growth and division and even communication among different cells. We furthermore suggest that such systems can be introduced into living cells for various biotechnology applications, such as switches for cell temporal and spatial manipulations.

## Key References

- MaityI.; WagnerN.; MukherjeeR.; DevD.; Peacock-LopezE.; Cohen-LuriaR.; AshkenasyG.A chemically fueled non-enzymatic bistable network. Nat. Commun.2019,10, 463631604941 10.1038/s41467-019-12645-0PMC6789017.^1^*This study shows how bistability emerges in reversible replication networks out of equilibrium and exposes the phase space of environmental conditions facilitating bistability.*
- WagnerN.; MukherjeeR.; MaityI.; Peacock-LopezE.; AshkenasyG.Bistability and bifurcation in minimal self-replication and nonenzymatic catalytic networks. ChemPhysChem2017, 18, 1842–185028112462 10.1002/cphc.201601293.^2^*In this work the bistable behavior is analyzed using theory and simulation, correlating the experimental initial conditions and reaction constants with the topology of bifurcation diagrams and the bistability operational zone*.
- MaityI.; DevD.; Cohen-LuriaR.; WagnerN.; AshkenasyG.Engineering reaction networks by sequential signal processing. Chem.2024, 10, 1132–1146.^3^*Two pathways are implemented in this work to control bistable networks rewiring through a “switch and erase” function and pathway modification*.
- MaityI.; DevD.; BasuK.; WagnerN.; AshkenasyG.Signaling in Systems Chemistry: Programing Gold Nanoparticles Formation and Assembly Using a Dynamic Bistable Network. Angew. Chem., Int. Ed.2021, 60, 4512–451710.1002/anie.202012837PMC798433733006406.^4^*This work describes cascade pathways, in which the switchable thiodepsipeptide bistable network outputs serve as input signals for consecutive processes that regulate features of Au nanoparticle shape and assembly*.

## 1 Introduction

The design and organization of man-made life constitute a long-standing target for chemistry and biology laboratories.^5−8^ Systems Chemistry research on this topic focuses on taking small steps toward the construction of unicellular systems, containing self-organized networks that feature three of the main characteristics of a living cell: (i) boundaries that fix the active molecules in specific settings, (ii) autonomous replication of individual molecules or molecular assemblies, and (iii) metabolism via the exchange of energy and materials with the environment.^9−11^ Clearly, in order to respond to changes in their immediate environment, the synthetic cells would need to receive and process signals that originate outside their borders, and integrate that information into a unified action plan. We propose that this can be achieved by mimicking signaling elements that regulate cell function by producing entities of codes with specific information, which in turn induce downstream activity.

**Figure 1:**
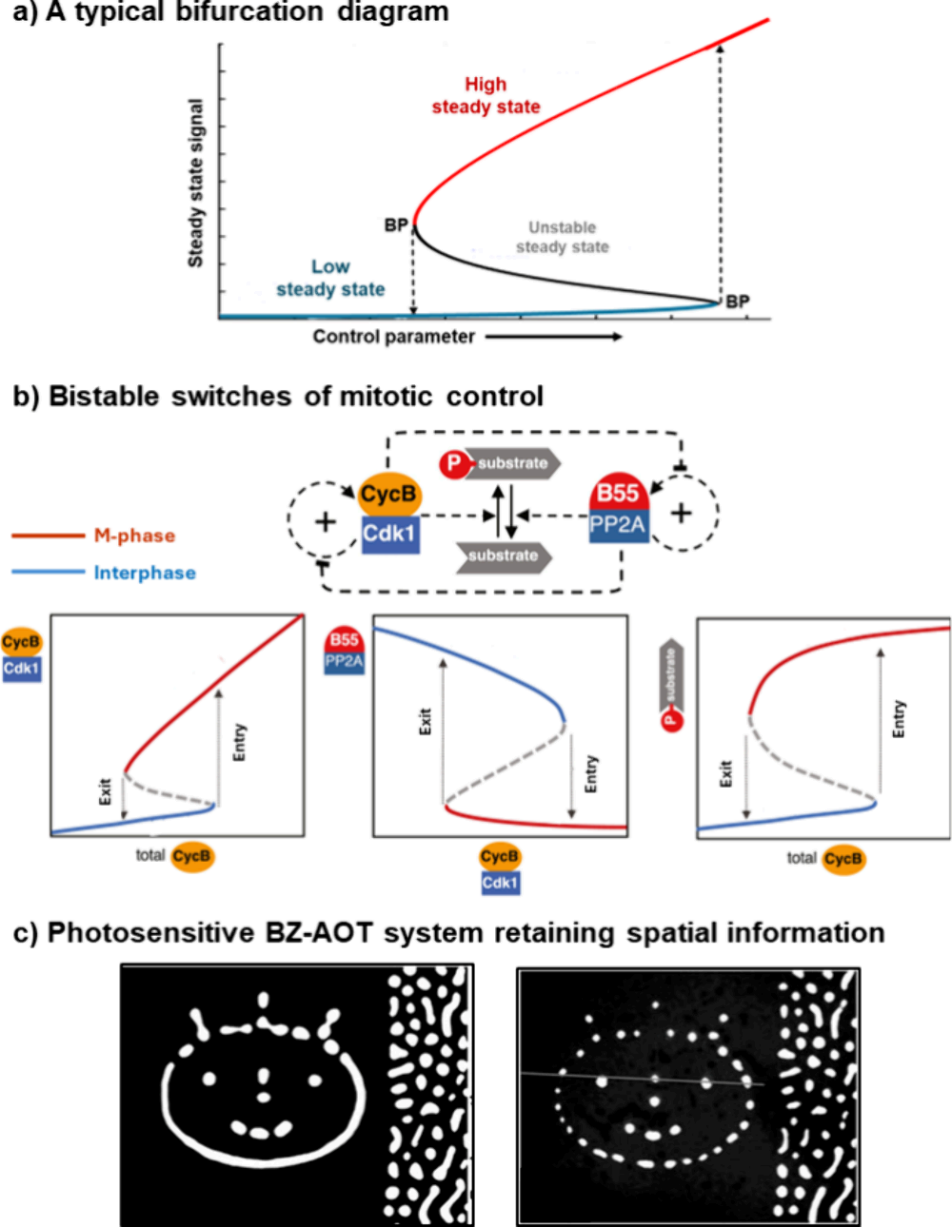
Bistability in biology and chemistry. (a) A typical bifurcation diagram. Two stable SS solutions are shown in *blue* and *red* lines, and an unstable solution is shown in *gray*. (b) Bistable control of cell division: mitotic substrate phosphorylation by interlinked kinase–phosphatase creating two distinct states, corresponding to interphase and M phase, and a switch function that prevents the cell from flipping back from the M phase to the interphase state.^20^ (c) A chemical reaction–diffusion memory device. The image taken shortly after decreasing the light intensity to within the bistable region is identical to a “face” mask (*left*), while after 1 h the image remained but the continuous lines transformed into dotted lines (*right*).^23^ Panel b Reproduced from ref (20). Available under a CC BY-NC-ND license. Panel c adapted with permission from ref (23). Copyright 2006 Wiley-VCH.

Analysis of cell responses to different cues, or the cell spatiotemporal organization during its life cycle, highlighted many examples where signaling is driven by bistable networks, that depending on their “history” occupy one out of two alternative long-lived steady states (SSs).^12−14^ Bistability is generated when the signaling network contains a positive feedback loop, such as autocatalytic motifs operating out of equilibrium, that drives switches with an all-or-nothing operation. The coexistence between two stable SSs is indicated by the occurrence of a hysteresis, as described by a bifurcation diagram showing how the SS of a particular variable as a function of a control parameter takes the form of an S-shaped (or Z-shaped) curve (Figure 1a).^15^ Numerous different control parameters are applicable for affecting the hysteresis function, including variations in the reaction component concentrations and reaction conditions (pH, temperature, salt additives etc.). In a range bounded by two critical values of the control parameter–marked BP for bifurcation points–the system admits three steady states, two of which are stable–marked *blue* and *red* for the low and high SSs, respectively–whereas the intermediate SS is unstable (*gray*). Large enough perturbations can cause switching between the stable states, while small perturbations may cause transitions between the unstable and stable SSs.

Bistable networks in cells yield long-term memory by converting transient signals or stimuli into sustained responses.^13,16^ The energy gaps between the alternative SSs of bistable switches give rise to the irreversibility of certain transitions in the cell cycle. For example, during the life cycle of bacteriophage lambda, upon infection of an *E. coli* cell, a choice is made between the lytic, virulent pathway that leads to cell death, and the lysogenic, dormant pathway which allows cell survival.^16−18^ Once chosen, the lysogenic state is extremely stable, however, a lysogenic cell may switch back to the lytic pathway, either spontaneously or due to induction by a cellular signal. Other well-known studies unveiled the molecular machinery that controls eukaryotic cell division, in which once a biochemical “decision” to proceed is made, the cell enters a qualitatively defined biochemical state, which makes the transitions from one cell cycle phase to the next phase irreversible.^13,19^ In preparation for chromosome segregation, when cells enter the M phase, multiple morphological changes occur, resulting in reorganization of cellular compartments. Such changes are driven by Ser/Thr phosphorylation of many protein substrates, predominantly by cyclin-dependent kinase 1 (Cdk1) in complex with cyclin B (CycB), where hysteresis of the mitotic switch emerges from mutual effects of Cdk1 and its counteracting phosphatase, PP2A:B55, on each other’s autoactivation loops (Figure 1b).^20,21^ Remarkably, this bistable switch is made of two distinct states, corresponding to interphase and M phase, without allowing the cell to rest in any intermediate transitional states. Furthermore, distinct thresholds for the mitotic entry and mitotic exit confer robustness of the M phase state and prevents the cell from flipping back to the interphase state.

The *de novo* design of positive or negative feedback motifs, and then more complex bistable or oscillatory systems remains challenging,^22^ primarily because the kinetic characteristics and energy aspects yielding such nonlinear functions are not always well-defined. Yet, several groups have shown that sustained bistable responses (i.e., self-organization into two alternately populated SSs) can be reached in synthetic systems. Like cellular bistable networks, the artificial prototypes are controlled by feedback elements, including autocatalysis and inhibition, and operate far from equilibrium by running in flow conditions or via constant chemical fueling. Significant progress has been made in studying bistability and multistability using small molecules, such as components of the BZ reactions and prebiotic thiols,^23,24^ peptides,^25,26^ gel materials affected by pH changes,^27,28^ and synthetic biology using nucleic acids, enzymes and regulatory transcription motifs.^29−32^ A pioneering example by Epstein has shown that a photosensitive BZ system dispersed in a water-in-oil microemulsion with a surfactant (BZ-AOT) can exhibit a rich variety of pattern formation, including localized structures. Snapshots taken from such experiments feature the images of a mask region in two interchangeable stationary states, observed upon illumination and after incubation for 1 h (Figure 1c).^23^ Bistability was confirmed by briefly increasing the light intensity to suppress the image, and then decreasing it back to within the bistable region, allowing the original image to slowly reappear.

Motivated by the role of protein assemblies and enzymes in signal transduction in cells, we initiated the investigation of synthetic coiled coil peptide-based bistable networks, driven by reversible autocatalytic reactions and continuously fueled by reducing agents that keep them active far from equilibrium.^25^ This review describes the progress made in exploring these bistable systems: (i) unfolding the design principle, reaction mechanisms, and dynamics that yield bistability, (ii) quantitative analysis of the bistable behavior for a large space of inherent, physical and chemical cues, (iii) implementation of various schemes of bistable switch concatenation to control downstream activity in homogeneous and heterogeneous environments. Toward the end we discuss how related systems can be adopted to yield bistability under flow conditions, expanded to exhibit multistability by coupling several replication networks together through competition for resource materials, and rewired toward oscillations. Furthermore, we elaborate on the relevancy of our findings for the origin of function in simple networks at early molecular evolution, and on the possibility to integrate the bistable networks into living cells for biotechnology applications.

## 2 Emergence of Bistability in Peptide Networks

**Scheme 1:**
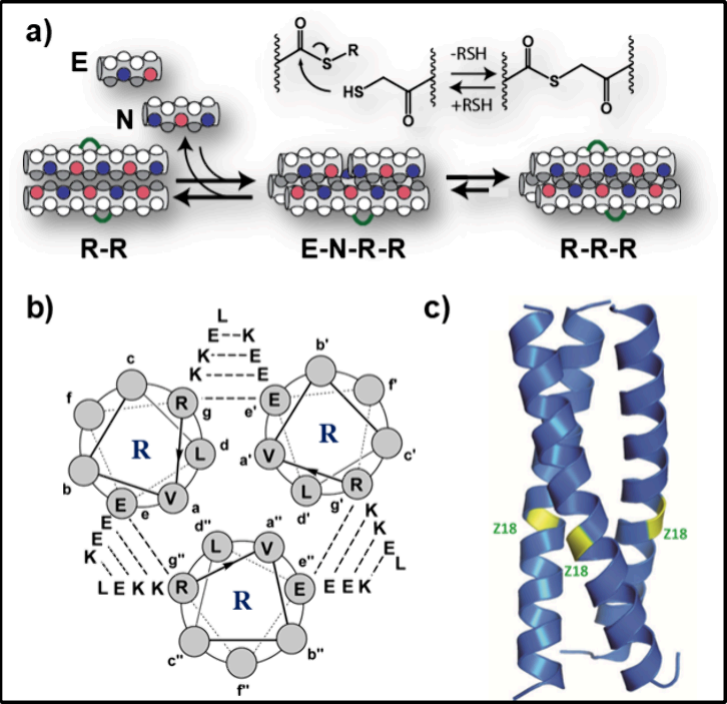
(a) A Reversible Replication Reaction Templated by the Thiodepsipeptide Coiled Coils. (b) Typical Helical Wheel Presentation of Amino Acids along the Coiled Coils Interaction Interface. (c) Crystal Structure of the Trimeric Thiodepsipeptide Complex; Z18 Marks the Thioester

Our bistable systems are propelled by reversible template-directed replication reactions of thioester-containing peptides (thiodepsipeptides; Scheme 1). Detailed analysis revealed the two prerequisites for bistability: (i) a disparity between the forward, autocatalytic pathway and the backward noncatalytic pathway, and (ii) continuous energy supply to keep the system far from equilibrium.

### 2.1 Reversible Replication Reactions

Nonenzymatic replication is accomplished by a single molecule or a small molecular complex that templates the proximal association and positioning of its building blocks to enhance their ligation, thereby forming a new copy of itself (Scheme 1a). The typical reactivity and chemical kinetics of different minimal replication systems have been previously reviewed.^10,33−36^ Our team, as well as others, applied coiled-coil peptides as functional motifs within networks, facilitating selective replication by control over structure, e.g., chirality, and function such as reciprocal catalysis, subnetworks organization, and simple computation.^37−43^

The sequences of the key thiodepsipeptide replicators (e.g., **R**; Scheme 1a) are derived from the GCN4-based peptide.^37,41,43^ This sequence contains a modification of a peptide bond to thioester, after replacing an alanine residue (A18) for a thioglycolic acid (Z18; Scheme 1c), and can hence undergo reversible formation-dissociation through thiol-thioester exchange reactions. The crystal structure of the thiodepsipeptide coiled coils shows a close similarity to that of the original peptide coiled coils.^44^ Like the all-peptide-bond prototype, the thiodepsipeptide enables the folding and simultaneous aggregation of stable complexes (Scheme 1b,c) that are held together by a Val-Leu hydrophobic core, and Lys-Glu salt bridges between residues on opposing helices. Sequences containing the Val-Leu hydrophobic cores form both dimer and trimer coiled coils,^45,46^ and both species are required for replication, since the template is a dimer, and the template-product complex is a trimer (Scheme 1a).

Initial characterization of reactions between the precursor thioester (electrophile) peptide **E** and a thiol-terminated (nucleophile) peptide **N** to form the replicators **R** (and release a small thiol molecule named **RSH** or **S**) revealed that product formation is governed by both the autocatalytic efficiency and the integrity of the **R** thioester bonds.^47^ Such ‘partial thermodynamic control’ over the replication has been proposed for the selection of replicators in the early molecular evolution.^48,49^ Indeed, analysis of product formation in small libraries, comprising the **R**-type replicators and their respective precursors, revealed that at early stages, far from the SS, the various replicators were formed in rates correlating with the relative efficiency of the template-assisted processes, while after some time, the concentrations of the efficient replicators continued to increase monotonically, and the less efficient replicators decomposed back to starting materials and reached SS at lower concentrations.^44,47,50^ Due to the reversible nature of the replication reactions, significant exchange-products amplification was also observed, enhancing the distribution of the more stable coiled coil thiodepsipeptides at the expense of the less stable ones.

### 2.2 Bistability

**Figure 2:**
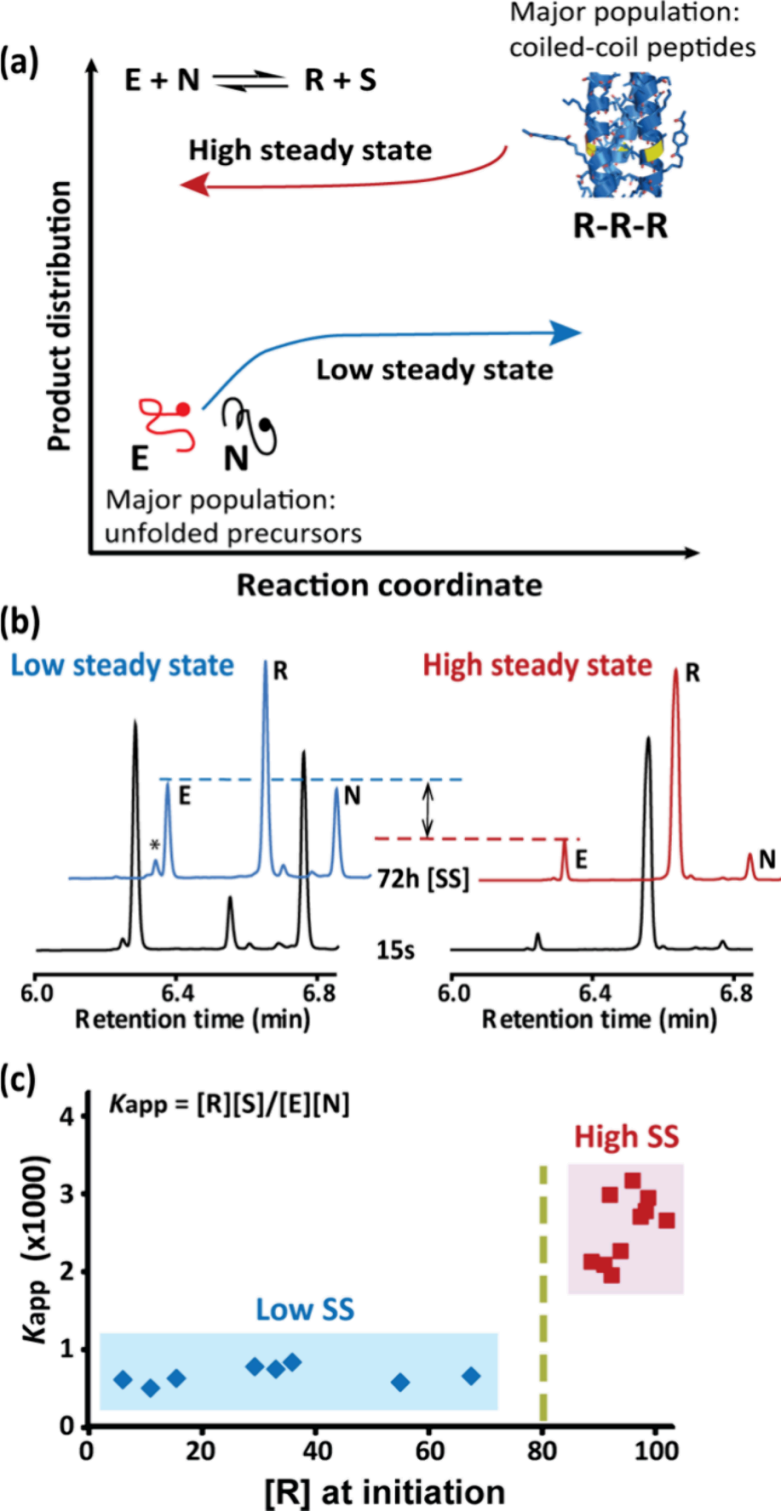
Bistability arising in thiodepsipeptide replication networks. a) A chemical reaction that invokes bistability. When initiated with high concentrations of the unfolded precursors **E** and **N**, the reaction network reaches the **Lss**, whereas initiation with a high concentration of the folded replicator **R** leads to a **Hss**. (b) UPLC traces obtained for the replication reaction at initiation and SS; reactions initiated either with 90 μM **E**, 90 μM **N**, and 10 μM **R** (*left*) or with 5 μM **E**, 5 μM **N**, and 95 μM **R** (*right*), leading to **Lss** and **Hss** product distributions, respectively. (c) Bistability diagram obtained by plotting *K*_app_ as a function of the initial [**R**]; in all cases, total peptide concentration [**E**] + [**R**] = 100 μM. Reproduced from ref (1). Available under a CC BY-NC-ND license. Copyright Gonen Ashkenasy.

The second-order autocatalytic reaction (Scheme 1a) constitutes positive feedback that leads to a nonlinear growth of the replicator **R**, and the formation of a coiled-coil structure by the product/template **R** renders it stable against the attack of peptide or small-molecule thiols, thus significantly slowing down its decomposition. These two mechanisms induce a significant disparity between the forward and backward reaction pathways, and consequently product distribution at the SS follows a clear bistable behavior. Bistability is experimentally established when multiple reactions are initiated with different concentration combinations of the substrates (**E** and **N**) and the replicator (**R**), while keeping their total concentration ([**E** + **R**]) constant (Figure 2).^1,25^ Reactions that are initiated with the substrates only, or the substrates and small amounts of **R**, reach a low SS (**Lss**), while reactions initiated with only **R**, or **R** and small **E** and **N** amounts, reach a much higher SS (**Hss**). We note the resemblance of this process to the lysis/lysogeny circuit of bacteriophage lambda (see Introduction, section 1), where the cell state is dictated by the availability and concentration levels of a single transcription factor (the lambda repressor).^17^ The observed product distributions are quantitatively characterized by *K*_app_ = ([**R**] × [**S**])/([**E**] × [**N**]), used for long-lived SSs, similar but not exactly the same as *K*_eq_ of the equilibrium reactions, and are clustered into narrow distinct regions for the **Lss** and **Hss** (Figure 2c). The existence of two distinct product distribution states (manifested by different *K*_app_) reflects that bistability is a network behavior and not a simple outcome of conformational changes of the replicator **R**. For some cases a narrow third “intermediate” cluster of SS *K*_app_ values is observed, residing in between the **Lss** and **Hss**.^25^ In this regime, the system is sensitive to small perturbations in the starting concentrations and is usually slow in reaching the SS.^27,51^

### 2.3 Energy Dissipation Driving Bistability

Significant progress toward the development of life-like systems was achieved via the design of transient structures and complexes that assemble due to chemical or light energy dissipation and are kept intact while fueling persists. Such transient assemblies can moreover feature secondary functions, such as binding small molecules or catalysis.^9,52−55^ Replication systems running far from equilibrium were also reported recently, yielding relatively complex behaviors, such as competitive or cooperative replication, selection of the fittest, and simple computation.^56−59^ Analysis of the thioester reversible replication kinetics shows that a continuously fueled reducing environment is crucial for bistability. Therefore, this system features a unique example of an elaborate function emerging within dynamic self-organized mixtures.

**Scheme 2:**
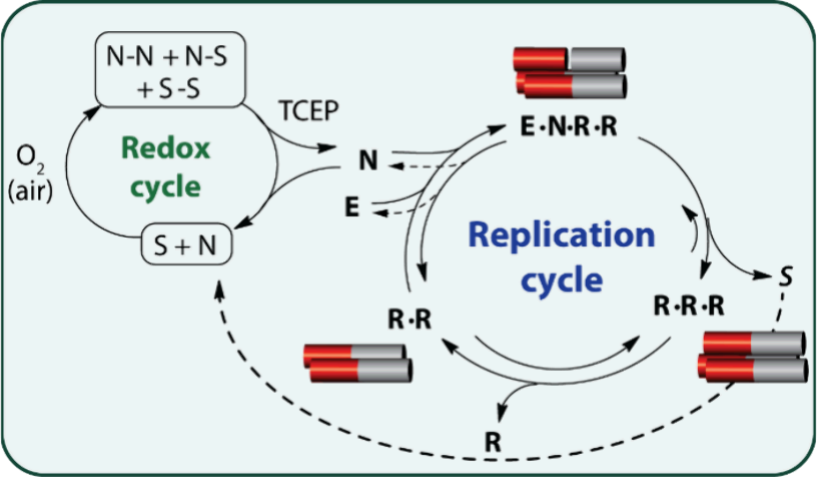
Coupled Redox-Replication Cycles Drive Bistability Continuous fueling by TCEP drives disulfide reduction in the redox cycle, facilitating reversible processes within the replication cycle. Reproduced from ref (1). Available under a CC BY-NC-ND license. Copyright Gonen Ashkenasy.

The bistable network topology is described in Scheme 2, highlighting coupling and energy dissipation between the redox cycle and the replication cycle. The redox cycle “engine” ran by excess tris(2-carboxyethyl)phosphine hydrochloride (TCEP) to reduce all the unreactive disulfide species into active thiols (**N** or **S**) that, in turn, drive reactions within the autocatalytic network yielding a positive feedback. Comparative experiments reveal that in the presence of TCEP (+Fuel), the network reaches one of two clearly distinct SSs, whereas in the absence of TCEP (-Fuel) the same network does not show bistability, but rather comes to an end at multiple resting states in linear correlation with the initial set of concentrations.^1^ The transient profile of the network SS behavior, and the energy-dissipation dependent behavior, are further supported by the fact that partial fueling–single-time fueling or initiation with low TCEP concentrations–lead to resting states that differ from the expected **Lss** or **Hss**, and moreover by that the system can be “revived” by proper refueling with TCEP to reach the expected SS positions.^1^

### 2.4 Pathway Modification Enabling SS Signal Transduction

The variable bistable behaviors, observed as a function of the applied combinations of reactants and products (Figure 1), hint for the possibility to design relatively complex signaling resembling biological pathways by yielding either the **Lss** or the **Hss** depending on the network’s history. Several previous studies disclosed multistep pathways that regulated supramolecular assembly and catalytic functions,^60,61^ but such motifs have rarely been incorporated for controlling self-organization and function of dynamic nonlinear networks.

**Scheme 3:**
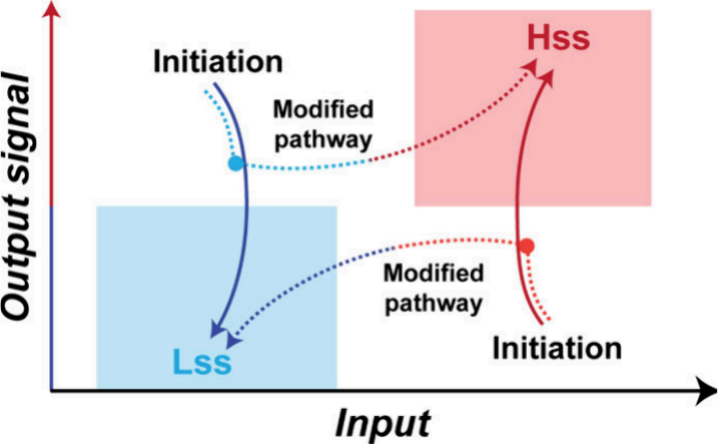
Signal transduction via pathway modifications The reaction pathway is altered through consecutive additions of the components while keeping the overall mass and energy limits unchanged.^3^

The pathway modification was experimentally achieved by modifying the order of addition of the network components along the reaction coordinate: consecutive additions allowed the reaction to cross the threshold separating the **Lss** and **Hss**, reflected by the unstable SS solution (Scheme 1a), thereby allowing the transduction of one type of signal into the other without changing the overall mass balance (Scheme 3).^3^ For example, the output of a control reaction mixture containing **R**, **E** and **N** that produces the **Lss** was compared with the output of a modified pathway manipulated by the two steps addition of the network components. The latter reaction is initiated with half the amount of **R**, while keeping the same amount of **E** and **N** as in the control experiments, and allowed to proceed until an “interim steady state” is achieved (4 days); thereafter, addition of the remaining half amount of **R** leads the system to the antagonistic signal **Hss**. The **Hss** to **Lss** modification is respectively achieved by comparing the one-step pathway generating the **Hss** from a mixture containing mostly **R** and low concentrations of **E** and **N**, with the two-step pathway, in which a reaction initiated with only **R** (**E** = **N** = 0) is allowed to proceed for 3 days, and then seeded with the “missing” **E** and **N**, to produce the **Lss**. Such pathway modification is applicable for a large set of reactions conditions, namely different concentration combinations and variable temperatures.^3^ We nevertheless note that “neutral” two-step modified pathways, which do not lead to changes in the SS signal type, are also observed for borderline cases in which the reaction components (**R**, **E** or **N**) do not cross the threshold point after the second addition, or in cases where the addition of the second amount is too early, i.e., before reaching the intermediate SS.

## 3 Mapping the Bistability Operational Phase Space

Like other out of equilibrium functions, bistability operates within a narrow space of conditions. Complementary theoretical, simulation, and experimental studies revealed the operational space for multiple inherent parameters and environmental conditions. The bistable zones are manifested by nonzero separation between the two SSs (Δ*K*_app_ > 0), while at the edges of the bistable zone a sharp phase transition leads to “ordinary” single SS behavior.

### 3.1 Theory and Simulation of the Thiodepsipeptide Bistability

The reversible nonenzymatic replication is modeled using the combined eq 1 that includes the substrate-template assembly (ENTT), templated ligation reaction (forming TTT + S), and dissociation of the product-template (TTT) to facilitate the next cycle.^2,62,63^ The template-assisted process is accompanied by a slower template-free reaction (not shown), and the total concentration of T in all of its forms is given using a single parameter *R* (eq 2). This model captures the second order autocatalysis and the disparity in forward and backward pathways as feedback motifs, thereby enabling the emergence of bistability.
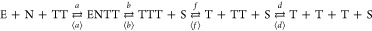
1
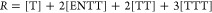
2The reaction rate is then described by
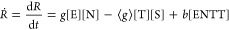
3where *g* is the template-free ligation constant (not shown) and *b* is the template-assisted ligation constant (eq 1).

At SS, this is simplified as
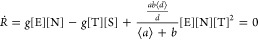
4

**Figure 3:**
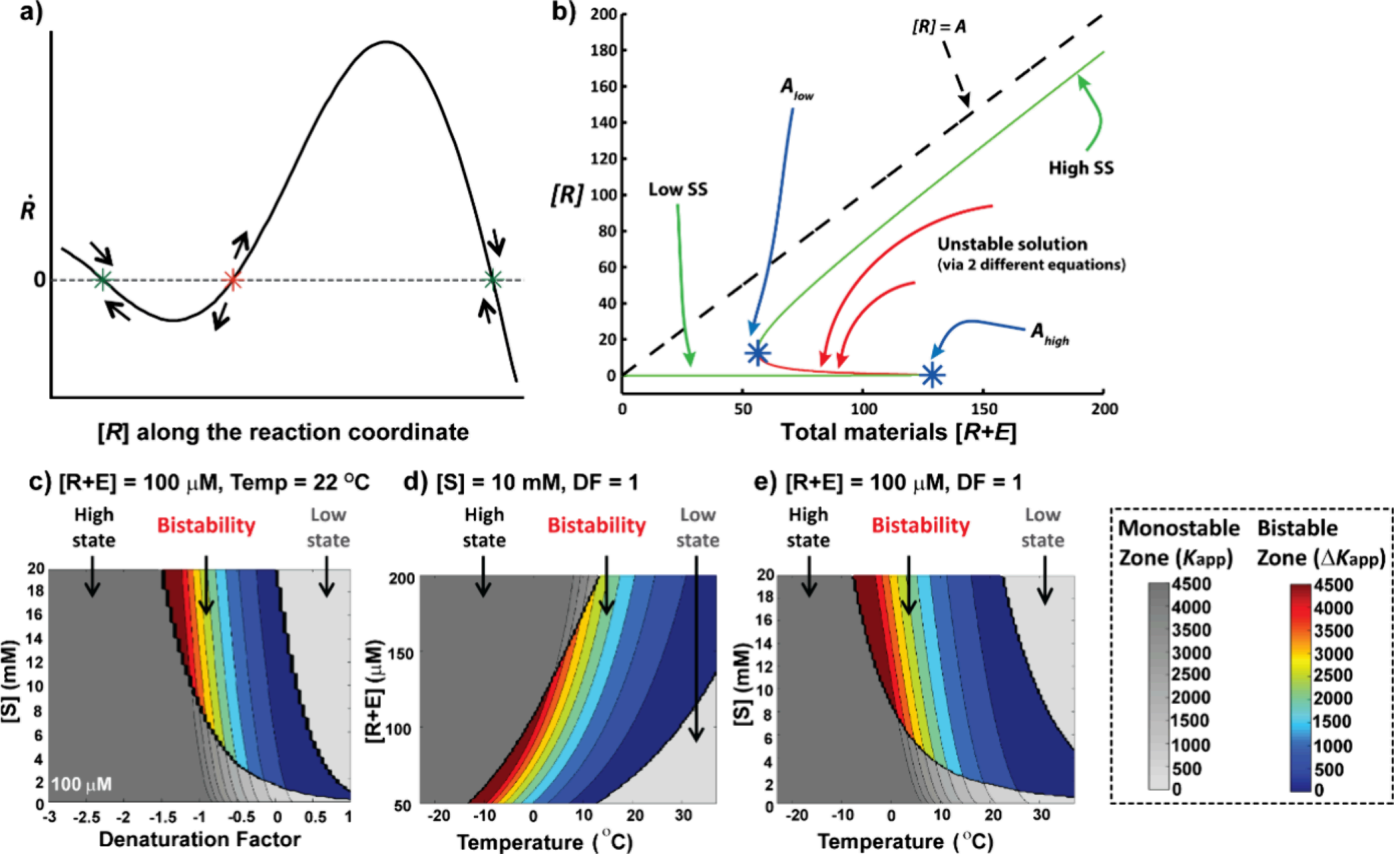
Kinetic simulation of the bistable system. (a) Reaction rate as a function of total template concentration; zero rates correspond to the SSs. (b) A generic bifurcation diagram; SS concentrations vs the total material [R + E]. (c–e) Contour diagrams showing the parameter space for low (*light gray*), high (*dark gray*), and bistable (*colored*) SSs. *K*_app_ values are shown for monostable (low or high) SSs, while Δ*K*_app_ values are given for the SS differences between the **Hss** and **Lss**. DF = denaturation factor. (a, b) Adapted with permission from ref (2). Copyright 2017 Wiley-VCH. (c–e) Reproduced from ref (1). Available under a CC BY-NC-ND license. Copyright Gonen Ashkenasy.

The appearance of bistability is clearly seen by plotting the time derivative of *R* as a function of [*R*] (Figure 3a), and in the bifurcation diagram that shows the SS values of [*R*] as a function of total material *A* (reactant plus product; Figure 3b). Figure 3a shows numerical computations for the reaction rates, where the rate zero values correspond to SSs, and the signs of the rate in the SS vicinity indicate whether they are stable or unstable. For this case, there are 3 SSs; the outer two points are the low and high stable solutions, while the inner point is an unstable SS solution. At lower or higher levels of total material concentration (outside the bistable zone), this analysis yields only one SS, and therefore do not display bistability.^2^ In addition to the typical bistable behavior, Figure 3b shows the analytical (closed-form) approximations of a stoichiometric upper limit for *R* (dotted line), SS curves (green and red), and the bifurcation points *A*_low_ and *A*_high_ (blue).

Using such computation analysis, we can calculate the bifurcation diagrams for any set of control parameters, yielding an overview of the bistability, and the locations of the SS solutions and bifurcation points.^1^ In order to better understand how the SS solutions depend on different combinations of experimental conditions, we map the bistability using 3D contour diagrams (Figure 3c–e). Each diagram shows the regions in parameter space corresponding to the low (light gray), high (dark gray), or bistable (colored) SSs. We plot the *K*_app_ values, or Δ*K*_app_ values for the bistable case, and show their dependence on thiol concentration, denaturation factor (DF), total material concentration, and temperature. In addition to highlighting that bistability forms under a wide range of parameter combinations, these theoretical studies disclose nominal *K*_app_ values for certain cases, threshold values needed to obtain bistability, and changes in parameter values that enable or disable bistability. Remarkably, since changing several parameters together may lead to compensation or cancelation, the system’s bistable behavior may be retained or otherwise rapidly abolished.^1^

### 3.2 Empirical Parameter Phase Space

**Figure 4:**
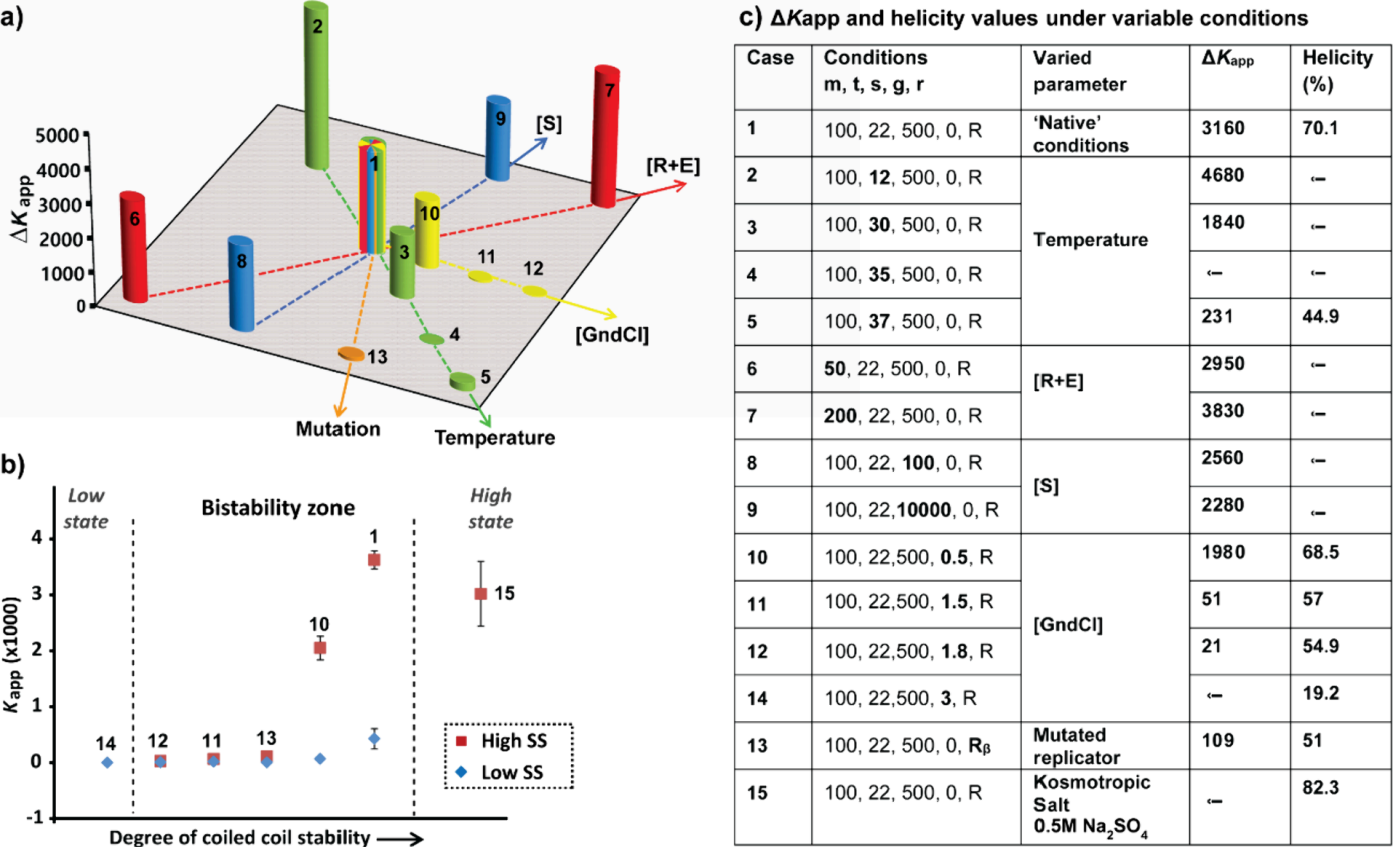
Environmental controls over the bistable network. (a) Bar graph presenting Δ*K*_app_ as a function of a specific perturbation in comparison to the “native” case Δ*K*_app_ (multiple colors). The *x* and *y* axes are not explicitly defined. (b) Experimental bifurcation diagram: *K*_app_ values as a function of the coiled-coil propensity. (c) Δ*K*_app_ and helicity values obtained under variable conditions. Numbers next to the data points in (a) and (b) depict the experimental conditions shown in this table, where m = total [R + E]/(μM); t = temperature (°C); s = [S]/(μM); g = [GndCl]/(M); r = studied replicator peptide.^1^ Reproduced from ref (1). Available under a CC BY-NC-ND license. Copyright Gonen Ashkenasy.

The theoretical study discloses useful guidelines for exploring the operational parameter space. Chronologically, our analysis started by investigating bistability under a “native” set of conditions that yield bistability with a switch point from the **Lss** to **Hss** at about 90 μM **R** (Figure 2). A two-dimensional bar graph from a large set of data (>220 runs),^1^ shows the Δ*K*_app_ values following changes in each of the studied parameters versus the native case (Figure 4a,c). An increase in Δ*K*_app_ is linearly correlated with the increase in total peptide concentrations, while a Δ*K*_app_ decrease is observed upon heating the mixture or after adding the denaturation agent. Using the Δ*G* = −*RT* × ln *K*_eq_ equation we compute the energy difference (ΔΔ*G* in kcal/mol) for each two states (using *K*_app_ ratios between the Hss and Lss) and show the dependence of the relative stabilities on the various experimental parameters. For example, we observed a linear dependence of ΔΔ*G* on temperature in the 12–30 °C degree range for various levels of thiol concentration (e.g., for entries 1–3 in Figure 3c). At one edge of the bistable zone, Δ*K*_app_ is significantly decreased by employing a thiodepsipeptide mutant that cannot replicate (***R***_**β**_; *orange* bar), or by further heating the mixture, yielding a single **Lss**. At the other end, a single **Hss** can be obtained by stabilizing the coiled-coil assemblies through the addition of a kosmotropic salt (Na_2_SO_4_).

Using the large set of data (Figure 4c), we recently addressed the challenging goal of reconstructing the entire operative bifurcation diagram (Figure 4b). The network dynamic trajectory is shown for various parameters that directly affect the self-assembly process–denaturant amounts, stability of different coiled-coil sequences, and (kosmotropic) salt additives. Like other “classical” bifurcation diagrams, the Δ*K*_app_ plot as a function of the degree of coiled coil stability (from CD; Figure 4c), displays a single **Lss** at unfolding conditions, passing through the middle bistable zone when folding affords efficient replication, and finally again reaching a single **Hss** for a too stable assembly that hinders coiled coils dissociation.^1^

## 4 Concatenated Bistable Switches Propelling Signaling Elements

As discussed above, crucial components of cell regulation are signaling elements that produce specific information and induce downstream activity. Molecular switches are driven by individual bistable networks, while more intricate intracellular signaling pathways are produced by sequential concatenation of several motifs.^20,64,65^ Specific connectivity facilitates the temporal and spatial control over function, since the exchange and recombination of individual motifs to form diverse pathways yields different final outputs depending on the cell’s chemical “history”. Several *in vitro* studies demonstrated sequential signal processing using nucleic acids, where the organization dynamics is regulated by “digital” base-to-base recognition and the rate of hybridization of complementary strands.^66−68^ For example, such systems disclosed efficient ways to create robust second-order behaviors and furthermore design DNA-encoded excitable circuits,^66^ and highlighted the design principles of regulatory networks that change their state in response to upstream stimuli to coordinate the timing of downstream signal expression.^68^ Concatenation of the peptide-based bistable motifs presents an important step toward application of additional biomaterials.

### 4.1 “Write and Erase” Using a Toggle Switch

**Figure 5:**
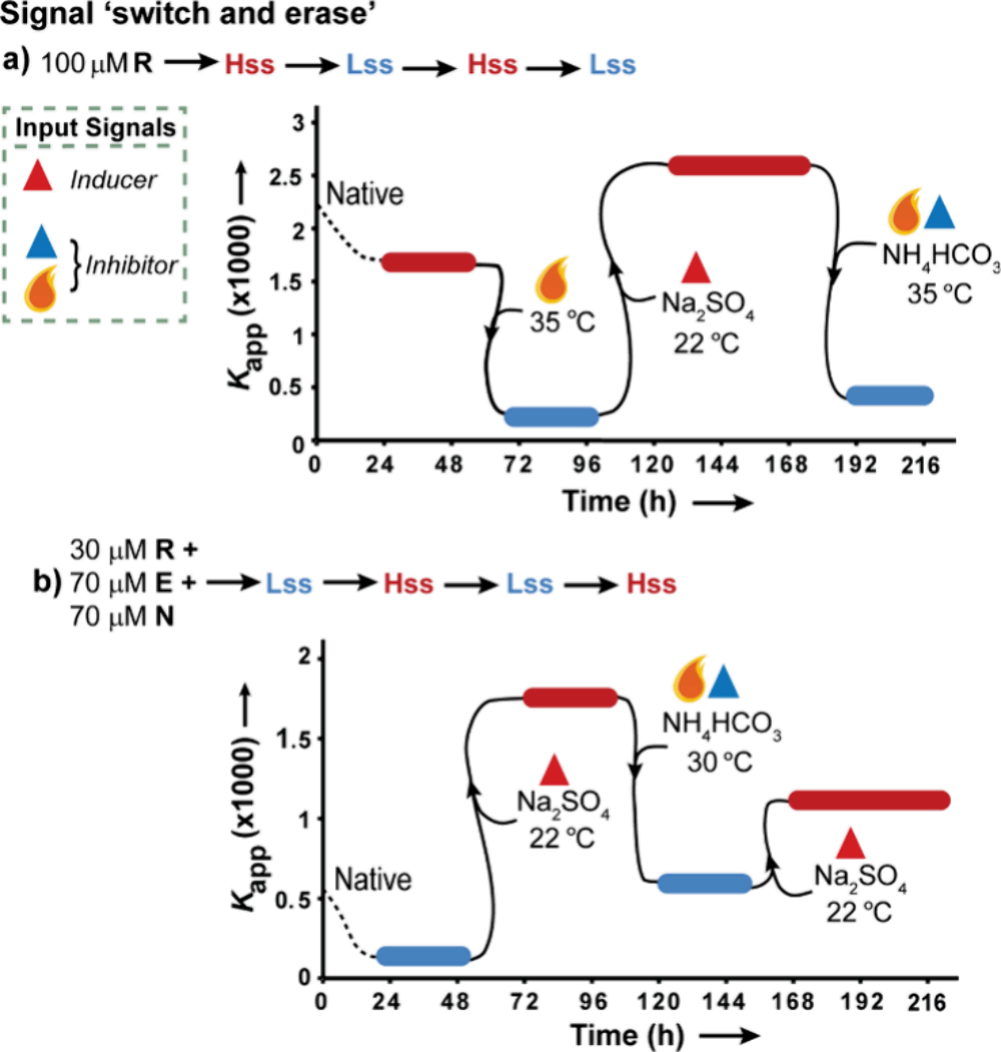
Continuous “switch and erase” functions. *K*_app_ values along the **Hss-Lss-Hss-Lss** (a) and **Lss-Hss-Lss-Hss** (b) signal processing. Modified with permission from ref (3). Copyright 2024 Cell Press.

Switching the bistable system between the two SSs was first demonstrated using temperature jumps to trigger the **Hss**-to-**Lss** switch, or by a two-step mixing procedure - first allowing **E** and **N** to react in the absence of the thiol small-molecule (1 day), then equilibrating the mixture in the presence of excess thiol–for the **Lss**-to-**Hss** switch.^25^ Based on the extensive data analysis that uncovered the network response to multiple control factors (Figures 3 and 4) it is possible to design concatenated toggle switches. A rather complex signal processing is presented by the “switch and erase” function, continuously manipulated in homogeneous mixtures. Here, via alternating application of chemical kosmotropic (Na_2_SO_4_) or chaotropic (NH_4_HCO_3_) salts, and physical constraints (up or down temperature jumps), the initial output signal (**Lss** or **Hss**) is switched to allow the system to reach a different SS, and then to revert back to the original SS (Figure 5).^3^ The reaction coordinate charts for four steps switches depict the initial concentration of reaction components, applied triggering at each step, time needed to reach the SS at each step, and the SS characteristics.

### 4.2 Bistable Control over Inorganic Nanomaterials

**Figure 6:**
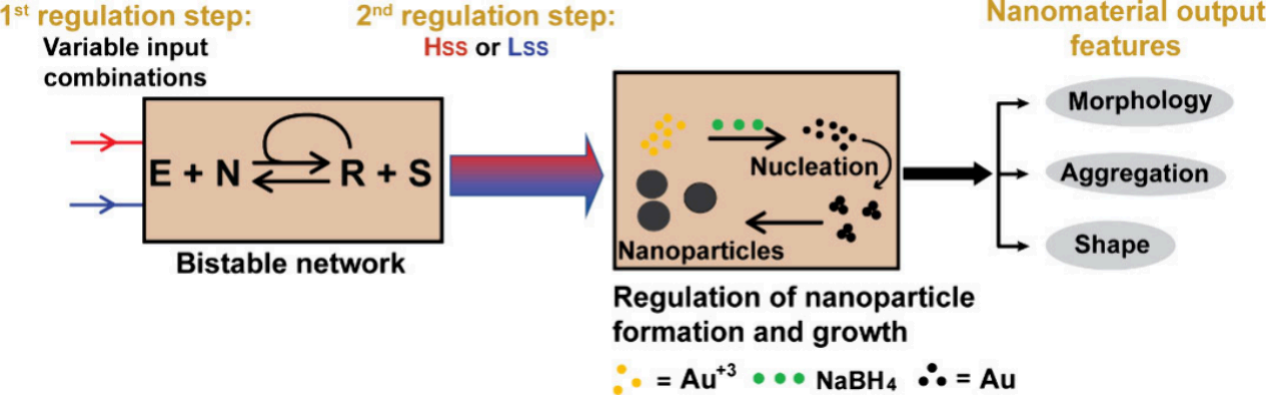
Design principles of the cascade reactions: the bistable network SS outputs are used as memory and regulate Au NP formation reaction and growth processes, in turn affecting the NP morphology, shape and assembly. Modified with permission from ref (4). Copyright 2021 Wiley-VCH.

Several synthetic bistable networks were previously applied for engineering chemical memory.^27,32,69,70^ After exploring the concatenated systems in homogeneous mixtures, we investigate whether the bistable SS output is a sufficiently general memory to control downstream activity under entirely different experimental conditions. Here, Au nanoparticles are chosen as the working model due to their diversity in shape, including spheres, and anisotropic nanostars, bipyramids, prisms, and rods. The bistable network **Lss** and **Hss** outputs serve as input signals for the consecutive nanoparticles formation reaction and growth (NRG) processes, which in turn, depending on the applied experimental configuration and reaction conditions, regulate various features of the NP shape and assembly (Figure 6).^4^ (i) Specific regulation of the NP morphology is evidenced when two different patterns, nanourchin and hyperbranched nanorods, are obtained after employing the **Hss** and **Lss** mixtures, respectively. The different assemblies evolved due to different degrees of nanoparticle stabilization in the two SS mixtures, each one possessing significantly different sets of interacting components (thiols, carboxylic acids, backbone amides, etc.). (ii) Analysis of the NP aggregation kinetics, by following the surface plasmon resonance signal (603 nm), indicates slower aggregation in the **Hss** mixture versus the **Lss** mixture. Remarkably, switching between the **Hss** and **Lss** (preceding section) leads to reversing the respective aggregation kinetics, featuring slower aggregation in the **Lss**-to-**Hss** switched mixture versus the **Hss**-to-**Lss** mixture. (iii) The shape-directing capability of the bistable network output is depicted when (spherical) Au NP seeds are allowed to cultivate in the two SS solutions, separately; significant shape retention is found in the **Hss** mixture, while incubation in the **Lss** mixture yields significant amounts of anisotropic architectures, including bipyramids and nanoprisms (which might have been kinetically trapped). We propose that the activation of such cascaded systems is a general phenomenon that provide guidelines for programming additional complex nanomaterials by signaling from aqueous mixtures.

## 5 Bistability Driving More Elaborate Dynamics

The insight gained from studying the bistable network dynamics and bistability in signal processing opens the opportunity to develop more elaborate functions. Natural progress includes running the replication systems in continuously stirred tank reactor (CSTR) setups, where the reversible replication SS output would be directly controlled by the applied flow rate. After modification and integrating controlled initiation and inhibition mechanisms, the network in CSTR can present oscillatory behavior. Furthermore, coupling two (or more) replication networks together through competition for resource materials would lead to the emergence of multistable behavior.

### 5.1 Nonenzymatic Replication Driving Chemical Oscillations

**Figure 7:**
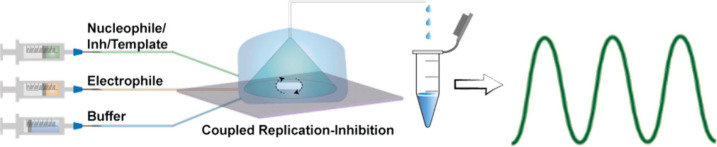
A general scheme describing the coiled coil replication reaction controlled by initiation and inhibition processes in a CSTR, featuring temporal oscillations in product formation. Modified from ref (78). Copyright 2023 American Chemical Society.

Rhythmic and oscillatory behavior drive many processes in living organisms, and synthetic oscillating reactions were also studied using small organic and biological molecules.^15,24,71−73^ In open systems like CSTR, the external fluxes direct the reactions to nonequilibrium SSs, which can be stable or unstable. In the case of instability, under specific controls, including concentration of the active materials and the environmental pH and temperature, one finds the oscillatory regime. We have recently applied the coiled-coil replication system and showed that the peptide assembly and specific interactions with the (**E** and **N**) replication substrates are crucial for sustained oscillations. Our exercise included the theoretical approach developed by Peacock-Lopez for oscillating reactions in continuously fed reactors with a ’sink’,^74,75^ and simulation of template-assisted replication and oscillations using experimentally realistic parameters.^76,77^ The experimental implementation further complies with the classical criteria for oscillations: (i) operation out of equilibrium (CSTR); (ii) controlled initiation of the replication reaction; (iii) efficient replication as positive feedback driving the oscillator to its highest point; and (iv) inhibitory negative feedback with a delay redirecting the system to a lower point, before expressing the next oscillation.^78^ The replication-inhibition network in batch mode produces a single damped oscillatory cycle, while these reactions in a CSTR evolve with reasonably regular and sustained oscillations (Figure 7). Future studies will highlight the design of coupled networks containing several oscillating peptides, on the way to producing autonomous protein-based circadian clocks.

### 5.2 Multistable Reaction Networks

**Figure 8:**
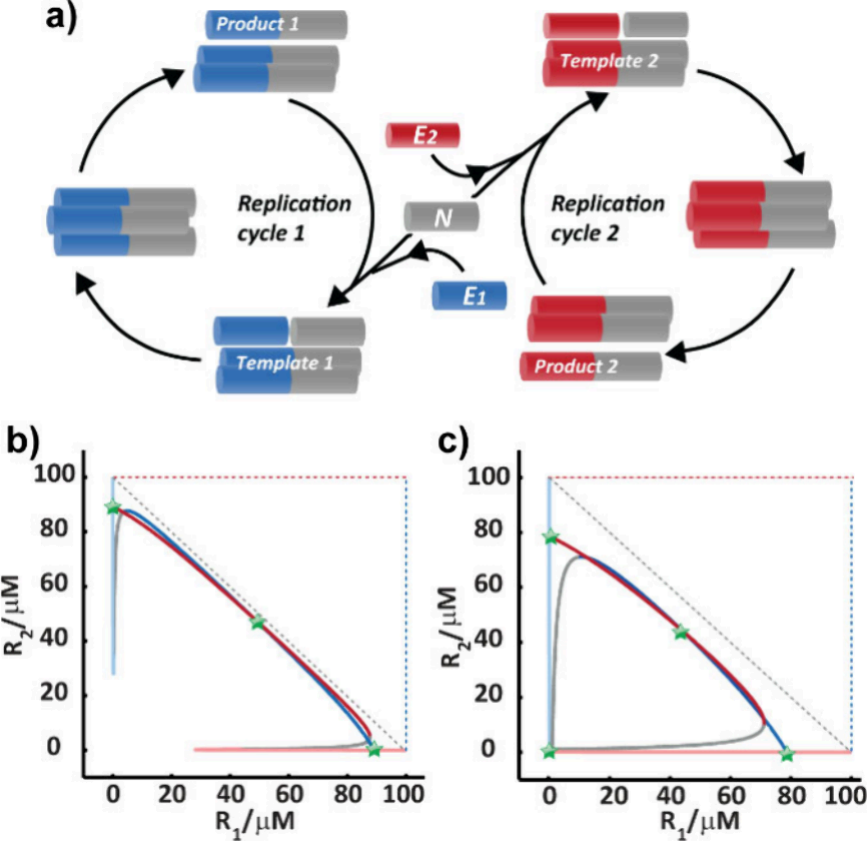
Multistable networks.^79^ (a) A schematic diagram of two reactions coupled through competition of **E_1_** and **E_2_** to react with the resource molecule **N**. (b,c) Multistationarity diagrams of two nullcline plots obtained for [**R_1_**] and [**R_2_**] replication, presenting three and four stable SS points (*green*), respectively. Modified with permission from ref (79). Copyright 2020 Wiley-VCH.

Merging two minimal bistable reactions into a unified network through competition for common resources leads to the emergence of multistability (Figure 8a). The unified reaction is driven by competition of the two electrophiles ***E*_*1*_** and ***E*_*2*_** to couple with one ***N***, forming two distinct products ***R*_*1*_** and ***R*_*2*_**. Although small, this system displays a wealth of interesting dynamic behaviors.^79^ Computational analysis yields the multistability map composed of two sets of nullclines (the curves where the rate of change of each variable is zero), reflecting the convolution of two different bifurcation curves. The curves are constrained by the total amounts of available reactants, and therefore any changes in initial reaction components or rate constants affect their size and shape. Topological changes that result from the intersections of two perpendicular null clines affect the number of SS points. This recipe leads to one, two, three or even four intersections of the stable sections of the bifurcation curves (Figure 8b,c).^79^ The respective experimental system is currently under investigation, where the main challenge is to form several product distributions distinctly separated from one another, beyond experimental data scattering.

## 6 Concluding Remarks

The enthusiasm for studying feedback loop systems, especially those enabling bistable functions and signaling motifs, originates from their centrality to the organization and dynamics of living systems. Our decade-long study now provides the physicochemical settings and guidelines for producing variable synthetic bistable chemical systems. While the results show a great deal of subtlety in the relationship between the reaction network conditions and its capacity to yield bistability (Figures 3 and 4), they also highlight the minimal requirements for bistability–positive (and/or negative) feedback mechanism and nonequivalent reaction pathways impaired by supramolecular structure formation. We propose that this simplicity bears implications for exploring the origin of protein functionality prior to the highly evolved replication-translation-transcription machinery.

Along the manuscript we have highlighted that the design of such systems, and their concatenation into signaling motifs, would benefit from quantitative analysis of the bistability operative regime and the energy differences between the alternative SSs. Indeed, our work has enabled the design of various signaling apparatuses featuring higher complexity than previously observed in chemical networks. Our current efforts are devoted to the incorporation of synthetic signaling motifs into artificial cells–either membranes containing or spontaneously formed coacervates–where the two (or more) states serve as memory and switch elements for controlling artificial cell growth and division, and even communication among different cells. Additionally, we hypothesize that our systems, or related networks, may be useful for chemical biology or biotechnology applications within native cells.
